# Stakeholder Perspectives on Power Transfer During Implementation of Psychiatric Self-Admission in Scandinavia: A Qualitative Study

**DOI:** 10.3390/healthcare14182909

**Published:** 2026-09-08

**Authors:** Maria Smitmanis Lyle, Alexander Rozental, Trine Ellegaard Laursen, Lena Flyckt, Inger Elise Opheim Moljord, Dag Øivind Antonsen, Merete Nordentoft, Sofie Westling, Rose-Marie Lindkvist

**Affiliations:** 1 Centre for Psychiatry Research, Department of Clinical Neuroscience, Karolinska Institutet & Stockholm Health Care Services, Region Stockholm, Norra Stationsgatan 69, 113 64 Stockholm, Sweden; alexander.rozental@ki.se (A.R.); lena.flyckt@ki.se (L.F.); 2 Department of Health, Education and Technology, Luleå University of Techonology, 971 87 Luleå, Sweden; 3 Department of Psychosis, Aarhus University Hospital—Psychiatry, 8200 Aarhus, Denmark; trine.ellegaard@rm.dk; 4 Nidaros Community Mental Health Centre, Clinic of Psychiatry, St. Olavs Hospital, Trondheim University Hospital, 7040 Trondheim, Norway; ingerelisemoljord@gmail.com; 5 Jeg Vil, Vi Kan, 7346 Oppdal, Norway; dag.oivind.antonsen@gmail.com; 6 Copenhagen Research Center on Mental Health (CORE), Mental Health Center Copenhagen, Copenhagen University Hospital—Bispebjerg and Frederiksberg, 2400 Copenhagen, Denmark; merete.nordentoft@regionh.dk; 7 Psychiatry, Department of Clinical Sciences Malmö, Lund University, 205 02 Malmö, Sweden; sofie.westling@med.lu.se (S.W.); rose-marie.lindkvist@med.lu.se (R.-M.L.); 8 Department of Psychiatry, Skåne University Hospital, 221 85 Lund, Sweden; 9 The ReLife Centre (Centre for Mental Health and Recovery Across the Life Course for Persons with Serious Mental Illnesses), Lund University, 221 00 Lund, Sweden; 10 Department of Clinical Neuroscience, Karolinska Institutet, 171 77 Stockholm, Sweden

**Keywords:** power transfer, empowerment, stakeholder perspective, qualitative, psychiatry, severe mental illness, implementation, self-admission

## Abstract

**Highlights:**

**What are the main findings?**

- Self-admission was understood in two contrasting ways: as a responsive and responsible approach that compensated for shortcomings in the mental healthcare system, and as an uncertain and potentially unsafe practice because it challenged professionals’ established control.
- Transferring decision-making power from professionals to patients creates tensions that shape how patient-centred and empowerment-oriented care is understood, accepted, and implemented.

**What are the implications of the main findings?**

- Successful implementation of patient-centred and empowerment-oriented interventions requires acknowledging and addressing the tensions associated with power transfer and responsibility between healthcare professionals and patients.
- Implementation strategies should focus not only on introducing empowerment in practice but also on communication efforts to develop a shared understanding of its purpose and address power asymmetries among stakeholders.

**Abstract:**

**Background/Objectives**: Psychiatric inpatient care is characterised by power asymmetries between care providers and care receivers. Despite international guidelines and policies promoting autonomy, involvement, and empowerment, these ideals remain challenging to implement in clinical practice. Psychiatric self-admission has been developed to strengthen autonomy; however, its implementation may challenge established power structures, professional roles, and responsibilities within mental healthcare settings. This study aimed to explore stakeholder perspectives on power transfer during the implementation of psychiatric self-admission in Scandinavia. **Methods**: A qualitative multi-source study was conducted using semi-structured interviews, focus group interviews, and document collection. Interviews were conducted with 36 participants involved in the development and implementation of self-admission in Denmark, Norway, and Sweden. Additionally, approximately 250 documents were gathered. The documentary material was analysed alongside the interviews and focus groups to provide an understanding of the implementation of self-admission models and how power transfer was represented within these processes. The analyses were inspired by methods in qualitative content analysis and document analysis. **Results**: Findings on stakeholder perspectives on power transfer during implementation revealed a tension reflected in two subthemes: ‘Responsive and Responsible’, where self-admission was viewed as a necessary response to needs that the healthcare system was unable to address, and ‘Unsafe and Unsound’, where self-admission was associated with uncertainties and risks related to losing control. **Conclusions**: Implementing psychiatric self-admission, which involved challenging traditional power relations in healthcare, was permeated by trust, distrust, and fear. The findings highlight the need to address stakeholders’ concerns related to safety and professional responsibility.

## 1. Introduction

Inpatient psychiatric care is a complex context within healthcare, balancing therapeutic support, risk management, and societal expectations regarding safety and suicide prevention [1]. Although psychiatric inpatient wards are intended to provide care in a safe environment during periods of severe mental distress, patients frequently describe experiences of limited autonomy, coercion, and hierarchical decision-making structures [2]. Despite worldwide ambitions to strengthen autonomy and address power asymmetries in healthcare [3,4], opportunities for empowerment and involvement in decision-making may be experienced as constrained in inpatient psychiatric care settings by healthcare staff due to the physical environment, power asymmetries, healthcare professionals’ attitudes, and concerns regarding patient safety [2]. Psychiatric inpatient care is characterised by asymmetrical power relations in which healthcare professionals hold authority over decisions related to admission, treatment, observation practices, and discharge [5,6]. Beyond formal coercive measures, psychiatric care may also involve forms of informal coercion, including persuasion, pressure, threats, and the use of authority to influence patient behaviour [7]. At the same time, healthcare professionals are frequently expected to prevent suicide and protect patients from harm [8], creating a clinical culture in which safety concerns may override patients’ perception of safety [9,10]. In Scandinavia, interventions such as self-admission to psychiatric inpatient care have rapidly spread in response to mental health policy and recovery-oriented frameworks, which increasingly emphasise autonomy, shared decision-making, and person-centred care [11,12,13,14,15,16]. Self-admission has been implemented across a large number of services in Denmark, Norway, and Sweden and involves allowing patients with severe mental illnesses, such as borderline personality disorder, bipolar disorder, and schizophrenia spectrum disorders, to admit themselves directly to psychiatric inpatient care when needed. Previous studies have suggested that self-admission may contribute to increased feelings of agency and security among patients [17], while also potentially reducing the need for coercive care [18], although this has not been confirmed in controlled studies [19,20]. Healthcare staff have described self-admission as meaningful and as a facilitator of more collaborative therapeutic relationships [21,22,23,24,25,26]. Previous studies of self-admission have frequently been conducted in specific patient groups and local healthcare contexts. Given the variation in self-admission models, caution is warranted when comparing findings across studies, highlighting the need for further research on the implementation and implications of self-admission in different contexts. Additionally, implementing self-admission within psychiatric inpatient care settings may affect professional roles, hierarchical structures, and traditional understandings of responsibility for suicide prevention and patient safety [27]. As self-admission entails transferring aspects of decision-making from professionals to patients, it provides a relevant context for examining how authority and responsibility are redistributed and negotiated in practice. Furthermore, the implementation of self-admission across several Scandinavian countries offers an opportunity to explore these issues across diverse yet broadly comparable healthcare settings. Scandinavian mental healthcare systems face several shared challenges, including the capacity and organisation of acute psychiatric services, the management of suicide risk and coercion, quality improvement in acute care, and the need for greater continuity and collaboration across services [28]. These common challenges provide an important context for the implementation of self-admission.

The relationship between professional responsibility and patient participation is often regarded as complex by healthcare staff, as increased patient autonomy and the right to admit oneself may at times challenge healthcare professionals’ clinical judgement. The Medical Research Council (MRC) guidelines for complex intervention highlight the importance of incorporating stakeholder perspectives during all phases of intervention development and implementation [29]. MRC (2021) [29] defines stakeholders as *“those who are targeted by the intervention or policy, involved in its development or delivery, or more broadly those whose personal or professional interests are affected (that is, who have a stake in the topic)—this includes patients and members of the public as well as those linked in a professional capacity”*.

There is still limited knowledge regarding stakeholders’ perspectives on power transfer in psychiatric inpatient care when interventions aiming to increase autonomy are implemented, and the concept of power transfer has not previously been explored in relation to self-admission. In this study, power transfer is understood broadly in line with Rowlands’ (1995) conceptualisation of power in empowerment processes, as involving the invitation of individuals into decision-making processes in ways that increase their capacity to influence structures traditionally characterised by constraints [30]. While person-centred care, shared decision-making, and patient empowerment all aim to increase patient involvement and influence, power transfer highlights the underlying redistribution of authority and responsibility that makes such involvement possible. In this sense, power transfer can be viewed as a fundamental dimension of person-centred approaches to care.

While previous qualitative studies with healthcare staff have explored experiences of self-admission among specific professional groups, mainly nurses [21,22,23,24,25,26], less attention has been paid to how the redistribution of authority and responsibility is negotiated and enacted during implementation. In line with Rowlands, an explicit focus on power allows examination not only of participation and influence but also of the structural and relational conditions that enable or constrain patients’ opportunities to exercise authority in decision-making processes [30]. Therefore, the aim of this study was to explore interdisciplinary stakeholder perspectives on power transfer, using the implementation of psychiatric self-admission in Scandinavia as a case.

## 2. Materials and Methods

The terminology used to describe individuals targeted by healthcare varies across the literature and includes terms such as patient, service user, client, care receiver, and individual. In this study, the term patient is used consistently for clarity rather than as a preference for one terminology over another.

### 2.1. Study Design

The study employed a qualitative design with an abductive analytical approach, using the collection of documents, semi-structured interviews, and focus group interviews with developers and decision-makers in Denmark, Norway and Sweden. Interview and focus-group data were analysed using qualitative content analysis [31], while documentary material was analysed using document analysis [32]. The combination of these approaches enabled the exploration of stakeholders’ understandings and experiences of power transfer while incorporating contextual information regarding the implementation of self-admission models. An abductive approach was considered suitable, as it allowed iterative movement between empirical observations and theoretical interpretation throughout the analytical process.

We used a broad definition of power transfer based on Rowlands’ understanding of power in empowerment processes, namely as inviting people into decision-making processes to release capacity and influence within structures that are normally characterized by constraints [30]. According to Rowlands, referring to ‘power’, rather than related concepts such as ‘participation’ or ‘influence’, will increase our ability to remain aware and deliberate in our understandings.

Reporting of the study followed the Standards for Reporting Qualitative Research (SRQR) guidelines (Supplementary File S1) [33].

### 2.2. Setting

The study was conducted within the context of psychiatric inpatient care in Denmark, Norway, and Sweden, focusing on self-admission in adult psychiatry between 2005 and 2025. Psychiatric care in these countries is characterised by publicly funded healthcare systems with a strong emphasis on the principle of need. A minimum of two self-admission models from each country were included, based on their establishment, standardisation, representativeness, and scalability within their respective healthcare systems. Model inclusion was discussed within the research group and informed by the co-authors’ collective knowledge and experience of self-admission implementation and evaluation across the Scandinavian countries. The included models were from the Capital Region of Denmark and the Central Denmark Region in Denmark; The Western, South-Eastern, and Central Regional Health Authorities in Norway; and Regions Skåne and Stockholm in Sweden. Details on the target group, structure and examples of formal power transfer in the studied self-admission models are presented in Table 1. These include patient involvement, the transfer of power in relation to the components of self-admission, for example contract content, the timing and length of self-admissions, and level of independence during self-admissions (administration of medication and assessments).

### 2.3. Participants

A purposive snowball sampling strategy was used to identify participants with experience within psychiatric self-admission. Inclusion criteria were individuals involved in the development, implementation, or evaluation of the selected self-admission models. The sampling strategy was chosen to capture perspectives from different stakeholder groups and organisational levels, including healthcare professionals, managers, researchers, and decision-makers. Participants were identified through published research articles, online searches, and the authors’ professional networks. At the end of each interview, participants were asked to suggest additional individuals.

Prior relationships between interviewers and participants varied. Some participants had no previous contact with the interviewers, whereas others had collaborated professionally with one interviewer before participating in the study.

Potential participants were contacted via email (n = 69), of whom 36 agreed to participate (27 women and 9 men), and are presented in Table 2. Individuals who did not participate generally did not respond to the invitation, and reasons for non-participation were not collected. Individuals with lived experience were represented among the participants. Due to the small size of these potentially identifiable subgroups, no further subgroup characteristics are reported. Recruitment continued until no further participants fulfilling the inclusion criteria were identified and the dataset was considered rich to address the study aim.

### 2.4. Data Collection

Both focus group interviews and individual interviews were conducted online through Zoom (Zoom Video Communications, San Jose, CA, USA) or in person, depending on the participants’ preferences. Interviews were conducted between April 2024 and May 2025, comprising 26 individual interviews and three focus group interviews. The focus groups consisted of three to five participants with diverse professional backgrounds and roles in the implementation of self-admission. Participants in the focus group interviews knew each other well and had previously collaborated during the implementation of self-admission. One individual participated in both an individual interview and a focus group interview. All participants received the same written and verbal study information, including information about the study aims and the researchers’ roles in the project.

An interview guide was developed by the author group informed by the MRC guidelines and included questions relating to views, experiences, and approaches to intervention design and adaptations, including factors influencing how self-admission was developed, implemented, adapted, and maintained (Supplementary File S2). Follow-up questions were used to clarify statements and encourage more detailed descriptions when relevant. While informed by Rowlands’ (1995) conceptualisation of power transfer [30], the interview guide was not structured around predefined questions about power transfer. Instead, participants were invited to reflect on their experiences of developing, implementing, and evaluating self-admission models.

Interviews were conducted by the first and last authors. The first author is a registered specialist nurse in psychiatric care and a doctoral student, and the last author holds a PhD. The first and last authors, both females with experience in qualitative research and research on self-admission, alternated as lead interviewer. No individuals other than the participants and the two interviewers were present during the interviews. Interviews lasted between 26 and 90 min, resulting in a total of 26 h, and were conducted in Danish, English, Norwegian, and Swedish without the need for translation. They were audio-recorded and transcribed in their original language using OpenAI Whisper large-v3 (OpenAI, San Francisco, CA, USA). Transcripts were reviewed and corrected against the audio recordings by the interviewers, who mastered Swedish, Danish, and English, and were able to understand Norwegian reasonably well. Repeat interviews were not conducted. Transcripts of focus group interviews were treated as a collection of individual statements. In addition, participants were asked to share documents they considered relevant to the development, implementation, use, or evaluation of the included self-admission models. This resulted in a total of approximately 250 documents, including policy documents, guidelines, PowerPoint presentations, meeting notes, grant applications, and emails.

### 2.5. Analysis

Data were analysed using an abductive approach involving iterative movement between inductive and deductive phases by the first and last authors working together. Emerging interpretations and preliminary findings were regularly discussed with the wider research team to support reflexivity, challenge assumptions, and refine the analysis.

First, interview and focus group transcripts were analysed according to the initial steps of qualitative content analysis [31] to identify meaning units, condensation, and coding. Transcripts were de-identified, and meaning units were highlighted, condensed, and assigned codes before being imported into the software NVivo 15 (Lumivero, Denver, CO, USA). Code development was discussed among the researchers to reach a shared understanding of the data. Differences in interpretation were resolved through discussion and repeated reference to the original data until agreement was reached. Subsequently, all data were interpreted and organised according to the six core elements of the MRC framework for complex interventions: context interactions, programme theory, stakeholder perspectives, key uncertainties, refinements, and economic considerations [29]. Data that did not fit within these elements were sorted into additional categories, including roles, reflections and learnings, and implementation activities. The MRC framework was used to organise and explore data rather than as a framework for generating findings.

Documents included a wide range of material, such as reports, presentations, meeting notes, investigations, correspondence, policy documents, and other records related to self-admission models, and were analysed following the first steps of document analysis [32]. All collected documents were first catalogued, including information on document type, origin, and context (Supplementary File S3). The documents were then imported into NVivo. The documents were reviewed systematically, and relevant content was identified and organised into the different categories alongside the interview and focus group data. Rather than serving solely as contextual information, the documents constituted an analytical data source that provided insight into decisions, events, challenges, discussions, and interpretations during the development and implementation of self-admission. They were treated as complementary data sources and analysed alongside interview and focus group data rather than being appraised according to document type, authorship, or intended audience.

During this stage, it became evident that issues related to power transfer recurred across data sources and stakeholder groups. This observation was not predefined by the analytical framework but emerged from the material during the analysis. Consequently, we returned to the data with an inductive focus on how authority and responsibility were understood, negotiated, enacted, and, at times, constrained in the development and implementation of self-admission models.

Documentary material, together with interview and focus group data, enabled a more comprehensive understanding of power transfer and implementation processes. The findings were subsequently interpreted through the theoretical lens of power transfer, informed by Rowlands’ conceptualisation of power within empowerment processes [30]. Rowlands’ conceptualisation informed the study’s understanding of power transfer but was not used as a coding framework. Through this iterative movement between empirical observations and theoretical interpretation, the final themes and overall interpretation were developed.

To maintain sensitivity to contextual differences, each national context was initially analysed separately. However, the analytical focus was directed towards identifying patterns related to power transfer across self-admission models rather than comparing countries.

Interview transcripts were not returned to participants for comments or corrections. However, member checking was conducted with six participants and used to strengthen credibility. Participants were given the opportunity to review and reflect on preliminary interpretations of their interviews, allowing for clarification, confirmation, and, where necessary, correction of the understanding. The feedback confirmed the researchers’ interpretations and did not lead to substantive changes in the findings. Confirmability was enhanced through reflexive discussions within the research team, including native speakers from all three countries and by ensuring that interpretations were grounded in the original data.

Coding and analysis were performed using the original-language transcripts to preserve contextual meanings and nuances. Quotations presented in the manuscript were translated into English after completion of the analysis. Initial translations were generated using ChatGPT version GPT-5.6 Sol (OpenAI, San Francisco, CA, USA) and subsequently reviewed and refined by the researchers against the original transcripts to ensure semantic accuracy and consistency.

### 2.6. Ethical Considerations

In Denmark, the Committees on Health Research Ethics concluded that formal ethical approval was not required, as the project was not considered health research, and the Danish Data Protection Authority raised no objections to the study being conducted. In Norway, the Regional Committees for Medical and Health Research Ethics (REK) considered the project to be a Swedish study and therefore stated that ethical approval should be sought in Sweden. The Swedish Ethical Review Authority (EPM) determined that the study did not require formal ethical approval (29 January 2024; Reference No: 2023-07705-01-01-517678). Data management was overseen by Lund University, Sweden. Participation was voluntary, and informed consent was obtained from all participants.

## 3. Results

Findings illustrate how power transfer, through the implementation of self-admission, was perceived as altering the existing healthcare system. In our analysis, we developed two themes representing perspectives on power transfer during the implementation of self-admission, where self-admission was perceived as Responsive and Responsible and as Unsafe and Unsound. On the one hand, the elements of power transfer during the implementation of self-admission were viewed as a necessary response to needs that the current system was unable to address, making changes to established work practices that appeared both justified and responsible. On the other hand, elements of power transfer during the implementation of self-admission were associated with perceived uncertainty and risks. Participants described a lack of understanding regarding power transfer, as well as how self-admission was intended to be put into practice. These contrasting perspectives highlight the tensions inherent in processes of power transfer. The themes are presented below together with representative quotes, to illustrate the main recurring findings.

### 3.1. Responsive and Responsible

Power transfer through self-admission was perceived as a response to needs that the existing healthcare system was unable to adequately address by trusting patients’ abilities. Participants described how the focus from the very beginning had been on the obligation to find solutions that could best support increased power for patients through autonomy, with practical details adapted to specific situations, emphasising the crucial role of specialist services for individuals suffering from severe illness with varying levels of capacity. In doing so, responsibility and authority for self-admission, based on patients’ own identified needs, could be transferred in a more manageable way.

“This is a vulnerable patient group with limited capacity to cope with stress that, for others, may seem trivial.” Document 64, Norway, Scientific publication 2008

“For some individuals, we set aside time to practice and learn how to use it. For others, it comes completely naturally, and they don’t experience any difficulties” Interview 13, Denmark

Giving patients access to self-admission was described as transferring responsibility to patients, involving a willingness to let go of the need to define the target group in advance and thereby avoid overly restrictive eligibility criteria. The importance of not limiting access based on criteria that, out of fear, would exclude large groups who could benefit from self-admission was emphasised.

“Some examples of challenges include: ‘Has the ability to control impulses’ (…) Many patients act impulsively (and may become threatening, aggressive, or violent toward themselves or others) when, for example, they are in a psychotic state in which they are frightened or feel forced to do things they do not want to do. This is part of the psychiatric condition itself.” Document 48, Sweden, Project overview [self-admission] August 2015

Healthcare staff had been motivated by introducing capacity perspectives into treatment, and even more so when experiencing how patients had been able to improve and take greater responsibility for their own course of treatment. Hence, the transfer of power from healthcare professionals to patients regarding how and when self-admission could be used contributed to improved care by making it both more flexible and predictable for patients.

Self-admission was described as a tool for creating a structure for beneficial collaboration, collective responsibility, and support between different parts of the system surrounding patients. Rather than psychiatric inpatient care being the entity in power due to its high status in the system as the provider of the highest level of specialist care, it was viewed as a support system for outpatient and municipal care. The role of specialist care was described as one that takes collaborative action and considers the contexts surrounding patients from a holistic perspective, rather than focusing solely on periods of severe ill health.

“The fact that we are actually also the closest people for some individuals is another matter. In those cases, it is reassuring that they have us on the ward when their immediate family members are exhausted or, for other reasons, are not present in daily life.” Document 66, Norway, Speaking notes for emergency psychiatric care conference

“For those who need a longer stay, we need to ask ourselves: is everything working on the outside? Does the patient have enough follow-up? How is the financial situation? How are they doing with their substance abuse? Is this good enough? That was our thinking all along: how can we help the patient in the best possible way. Not a thought about hospital misuse.” Interview 27, Norway

Participants reflected on ethical dilemmas arising from power transfer through self-admission when the trust transferred to patients’ abilities and decision-making power was withdrawn during periods of severe deterioration, and on the responsibility to carefully consider its potential consequences for the patient.

“You are allowed to admit and discharge yourself, and suddenly you get really ill, and must be put on restraint. Is that breaking the trust?” Interview 4, Sweden

### 3.2. Unsafe and Unsound

Participants described a perceived resistance among physicians to the transfer of power to patients, motivated by concerns that increased patient autonomy would require physicians to approve decisions perceived as potentially harmful.

“The idea is that patients should learn to take more responsibility by being offered the model—you should get a different perspective of your illness and all that. But I think many of the doctors were afraid that it would end up forcing them to make difficult decisions, or that you would be forced to approve bad or destructive decisions from the patient because they are self-admitted” Interview 11, Sweden

“Is it professionally acceptable for the patient to come on their own initiative and not be assessed by a physician? Can the senior physician support and accept this? Is there a sufficient level of safety regarding medication, risk of self-harm, suicidality, and dangerousness?” Document 68, Norway, PowerPoint presentation during visit by the Danish Capital Region

Concerns were expressed that patients who were given greater autonomy in relation to self-admission, including self-administering medication based on their own perceived needs and undergoing fewer assessments by healthcare professionals, might engage in self-harm or attempt suicide during a self-admission period. Questions were raised about a potential mismatch between the transfer of decision-making power to patients and the continued accountability of healthcare services for outcomes. Healthcare staff were described as having limited confidence in the concept, driven by fear about self-administered medication, increased patient autonomy, and the potential for misuse, while physicians remained ultimately responsible for patients’ care.

“There were not many staff members who liked it or believed in it at the time. And the main concern was that patients would bring their own medication and make their own decisions and manage things independently. There was a great deal of fear surrounding all of that.” Interview 26, Sweden

“During the physicians’ meeting, the physicians expressed strong concerns about the responsibility placed on them, despite the fact that they may not see the patients at all during the admission” Document 72, Sweden, Meeting notes 2018

The transfer of decision-making power over the use of inpatient care from healthcare services to patients was described as being associated with fears of adverse events. These concerns were perceived to have an emotional impact on healthcare staff and the responsible physician, contributing either to the discontinuation of self-admission and resistance to its reimplementation in wards that had experienced a serious adverse event, or to clarifications of procedures regarding medical assessments during self-admissions.

“Reasonable precautionary measures should have been taken, such as a more in-depth conversation with the patient, and possibly a suicide risk assessment conducted by a physician, as well as securing any medications brought by the patient (…). Finally, at an overarching level, it is noted that the specialist physicians at the unit had previously raised concerns that [self-admission] (…) involved potential safety risks. Among these risks, the issue of responsibility and accountability was specifically highlighted.” Document 155, Sweden, Socialstyrelsen

“Clarification from the Norwegian Directorate of Health. The psychiatrist on duty should be informed when self-admission takes place. The psychiatrist can provide advice and guidance or provide assessment. All patients admitted to self-admission must be assessed by a specialist during the inpatient stay.” Document 27 Norway, Visit from the Capital Region of Denmark’s Psychiatry Services, PowerPoint presentation 2016

Some participants expressed concerns about the consequences of adverse incidents, reflecting on how such events might have been interpreted differently in interventions involving a transfer of power to patients. Their reflections suggested a perception that the early implementation had been fortunate in avoiding serious incidents, as such events might otherwise have jeopardised continued implementation efforts.

“We had many years without incidents, and to make a U-turn when something happens, it’s not like you stop with other treatments (when incidents happen)” Interview 18, Sweden

“We did not have any incidents that made us feel that this would not work. Perhaps we were lucky, as accidents or suicides can always happen.” Interview 24, Norway

The concerns surrounding adverse events also appeared to be intertwined with broader issues of power and authority. Participants perceived safety issues as sometimes functioning as expressions of underlying power struggles.

“Indirectly, of course, there were a lot of issues of power. Who decides what, and patients can’t decide for themselves. Even though they never said it, they hid it behind patient safety issues” Interview 4, Sweden

The healthcare system was perceived as a hierarchical system unwilling to change and let go of power. While individual healthcare professionals may support participation within their own practice, sustained change in healthcare was perceived as dependent largely on culture and systems rather than on individual action alone.

“Some healthcare professionals may, on their own and as part of their regular work, place extra emphasis on the patient’s wishes/goals and decisions. However, the effect is limited to interactions with that particular professional, and otherwise the patient must relate to staff with different attitudes.” Document 246, Norway, e-mail correspondence

Participants described how self-admission was difficult to implement due to perspectives suggesting that self-admission *“undermines the established division of responsibilities between primary care and specialist healthcare services”* (Document 64, Norway, Scientific publication 2008). They described perspectives on self-admission leading to changes that undermined the concept due to refusals to let go of power in systems characterised by distrust. Several participants described the establishment of specific rules and boundaries to prevent misuse of the service, thereby increasing acceptance of self-admission among sceptical healthcare professionals. These concerns were often rooted in the belief that transferring the power to self-admit to patients would result in frequent or excessive use of inpatient care.

“The patients were not given immediate access to admission/the bed but instead had to wait up to one week before their initiative was accommodated. I understand it as though this adjustment undermined the project. The whole point of the project was to examine what would happen if patients could manage admissions themselves. Instead, the patients still encountered the institution’s boundaries and limitations. The patients did not experience receiving treatment/admission when they themselves considered it necessary.” Document 246, Norway, e-mail correspondence

Stakeholders argued that similar methods were already in use or had been used previously, questioning what was new or special, missing out on the point that, in prior initiatives, professionals were still in complete control and power transfer was lacking.

“That was the difficult part, I think, for the staff (…). Understanding that they tended to think, ‘Well, we already have planned admissions, it’s the same thing.’ But no, it’s not. Because with planned admissions, there was still always this element of assessment by psychiatry, or this element of selecting and hand-picking patients. Here, that element doesn’t exist.” Interview 19, Sweden

In light of the transfer of decision-making power to patients, self-admission was at times described as a form of “luxury care”. Opponents had expressed concerns that self-admission would be overused for inappropriate reasons or as a hotel service rather than for legitimate clinical needs, misunderstanding the underlying idea: ‘Is this even healthcare?’.

“The ones who were against the project said that they (the patients) would just use it like a hotel, misuse the possibility and being admitted all the time. The people who had that kind of thinking even before the project, kind of kept having it”. Interview 10, Denmark.

The paradox of healthcare professionals simultaneously occupying positions of authority while perceiving their own contributions to care as being of limited importance to patients was described as particularly striking. Participants reflected on how the transfer of power to patients could be experienced as a loss of professional authority, generating uncertainty regarding professional roles. Such perceptions were described as potentially leading to an abdication of responsibility and reduced engagement with self-admission as a consequence of the perceived loss of power.

“Physicians have distanced themselves from patients admitted through [self-admission], despite the nursing staff expressing a need for their involvement, this has created conflicts regarding who is responsible” Document 72, Sweden, Meeting notes 2018

## 4. Discussion

In this study, the empirical example of the implementation of psychiatric self-admission in Scandinavia was used to explore stakeholder perspectives on power transfer. In the context of severe mental illness and psychiatric inpatient care, the implementation of self-admission challenged the existing healthcare system by transferring power and responsibility from healthcare staff to patients. On the one hand, this shift was perceived as both necessary and responsible, as self-admission was understood to address important shortcomings in current healthcare practices while also accommodating different individual needs and capacities. On the other hand, the same shift was simultaneously associated with concerns about risks, uncertainty, and potential negative consequences and was therefore experienced as challenging for those involved, leading to power struggles and the abdication of responsibilities, perhaps creating an even less safe environment. Two key concepts run through our findings on stakeholder perspectives on power transfer from healthcare professionals to patients: trust and fear.

The view of power transfer as essential and responsible aligns with previous studies emphasising the importance of more equal relationships in mental healthcare settings. In line with our findings, involving patients in decisions about their care is perceived to be both ethically desirable and consistent with person-centred care [34,35]. The trust that underlies the transfer of decision-making power from healthcare providers to patients requires that healthcare professionals have confidence in the autonomy and ability of patients to act in their own best interests [36]. Increased patient involvement in healthcare improvements is dependent on the professionals’ mindsets [5]. However, in our findings, the perceived loss of authority highlights concerns regarding risks, particularly among physicians, in relation to power transfer. This reflects an emphasis on safety and ethical clinical responsibilities, which may imply a lack of trust in others, and divergence between professionals’ perceptions of safety and patients’ own experiences. As pointed out by Rowlands (1995), empowerment processes require professionals to act as facilitators who have respect for and confidence in those targeted and as such cannot expect to control outcomes if empowerment is authentic [30]. Although some participants interpreted references to patient safety as a barrier to power transfer, our study cannot determine the underlying reasons for these concerns. Instead, the findings suggest that issues of safety, responsibility, and power transfer were closely intertwined during implementation. This may partly reflect the responsibilities associated with medical accountability, risk assessment, and patient safety. Healthcare professionals responsible for clinical decision-making may experience tensions between supporting patient autonomy and maintaining responsibility for the consequences of care decisions. These considerations highlight how power transfer may be interpreted differently depending on role and responsibility. Additionally, patients have described an increased sense of safety within the psychiatric inpatient setting when having control over decision-making regarding their care [37], revealing that what is considered safe from a clinical perspective may not necessarily align with how safety is perceived by patients. Future research could explore such differences between stakeholder groups in detail.

Some perspectives on self-admission challenged the concept by reflecting an unwillingness to let go of power. This may not be new, as paternalistic practices have been shown to dominate healthcare, particularly psychiatry, for decades despite an increasing emphasis on autonomy [6]. Healthcare staff in psychiatric settings often describe taking responsibility for patients’ well-being in ways that may limit involvement [38]. This tension may help explain why physicians experience a sense of losing control while remaining accountable in relation to self-admission, when power over admissions, medication, and, in some models, even assessment, is transferred to others, while the sense of responsibility for the outcomes remains with the physician. Changed power dynamics in mental healthcare may create clashes with existing medical systems, as has been demonstrated in other research on a shared decision-making model [39]. This notion of a reconfigured power structure may be even more apparent in the studied context of inpatient psychiatric care and severe mental illness. Our findings indicated that physicians experienced a perceived fear of being required to approve potentially destructive decisions when patients were self-admitted. At the same time, previous research involving patients has shown that self-admission was used as a strategy to prevent destructive behaviours [40]. This suggests a potential discrepancy between physicians’ concerns regarding self-admission and patients’ motivations for using self-admission.

Our findings point towards changed views on safety when transferring power from healthcare professionals to patients, indicating that serious adverse events are perceived as less acceptable in interventions such as self-admission than in other interventions. This raises questions about whether implementation of some interventions needs to be conditional on the absence of serious incidents, and whether this is reasonable, responsible or problematic. Furthermore, our findings point to the need for healthcare professionals to feel safe and that power may not truly be transferred unless let go of willingly and with confidence. Also, as Rowlands (1995) highlights, formal power transfer, such as providing opportunities for decision-making and self-assessment, is no guarantee of a perceived ability or entitlement, both of which are necessary for empowerment [30].

Rowlands (1995) [30] describes empowerment as a process in which the professionals involved need to understand how an intervention affects the people involved. This understanding needs to be considered when planning implementation and evaluation, as the intended outcomes of self-admission may not be achieved if the meaning and purpose are interpreted differently by stakeholders [30]. In our study, participants described how self-admission was at times misunderstood by stakeholders, suggesting that its purpose and underlying principles were not clearly communicated or shared. Such misunderstandings may limit opportunities for meaningful engagement with self-admission and reduce its potential to promote empowerment. Additionally, in a previous study, patients described how inpatient staff often lacked knowledge of the personal strategies and work they had developed in outpatient care, and how this limited healthcare professionals’ ability to provide individualised support during self-admission [41]. In line with Rowlands (1995) [30], our findings indicate that successful implementation of empowerment in practice requires more than introducing a new method. It requires cultural change and that healthcare professionals, decision-makers, and other stakeholders develop a shared understanding of the aims and values, including establishing criteria for evaluating the empowerment process [30]. Involving people with lived experience throughout implementation and evaluation will further support the communication of the needs and perspectives of those it is intended to benefit.

On a final note, whether considered unreasonable or appropriate, the degree of power transfer during the implementation of self-admission can be reflected upon in the greater scheme of things.

“If the psychiatry designs the contracts and offers them to individuals, what exactly is self-decided?” Document 49, Sweden, PowerPoint presentation

### Methodological Considerations

Previous studies on stakeholder perspectives on self-admission from the perspective of healthcare professionals have often relied on frontline staff, where there is a risk of participation bias in the sense that individuals with more favourable views of self-admission may be more likely to take part. This study included decision-makers and individuals involved in the implementation process, making it possible to capture a range of perspectives grounded in their broad experiences.

Although perspectives from individuals with lived experience were represented in the dataset, the study was not designed to compare stakeholder groups. Furthermore, the small number of participants within each role limited the extent to which these perspectives could be presented separately without risking identification. As participant characteristics were reported at an aggregated level to protect confidentiality, it was not always possible to distinguish whether specific findings reflected professional, national, or model-related differences. Consequently, what may appear to be a professional perspective could partly reflect characteristics of a particular national context or self-admission model. In addition, some findings were informed by documentary material and by participants’ descriptions of stakeholders’ perspectives. Thus, certain accounts should be interpreted as representations of perceived positions and concerns rather than as direct accounts from the stakeholder group concerned.

The study included an unequal distribution of participants across the three Scandinavian countries, with an overrepresentation of Swedish participants. This was likely due to the interviewers being Swedish, as well as a current national interest in self-admission at the time of the study, making it easier to recruit participants. Despite our efforts to recruit participants from different stakeholder groups, organisational levels, and national contexts, some relevant perspectives may not have been captured. Furthermore, the uneven distribution of participants across countries may have influenced the prominence of certain experiences and viewpoints. Although efforts were made to include a broad range of stakeholders, our recruitment partly relied on professional networks and participant recommendations. Consequently, the study may still have been influenced by participation bias, and some perspectives may not have been captured.

Interviewing co-authors and using member checking were strategies decided upon from the beginning and were prerequisites to achieving the study aim. The trustworthiness of the findings is strengthened by the systematic approach used during the analysis and by the collaborative discussions throughout the analytical process. Our qualitative findings are influenced by our interpretations and contextual understanding. Although reflexivity and continuous dialogue within our research team, including a co-researcher with lived experience, were used to support confirmability, alternative interpretations of the data may exist. All members of the research team had prior experience of developing, implementing, and/or evaluating self-admission models. In line with reflexive qualitative research, we viewed the researchers’ positions, our positions, as sometimes outsiders and sometimes insiders, not as something that could be eliminated from the research process but as factors requiring ongoing reflection and transparency [42]. Throughout the study, we paid attention to how prior experience, professional involvement, and assumptions regarding self-admission could shape both data collection and interpretation. Six participants were also co-authors of the manuscript. They were included because they fulfilled the study inclusion criteria through their involvement in the development, implementation, and/or evaluation of self-admission. Several of the interviewed co-authors were suggested by other non-author participants. Several co-authors participated in the analytical process, including analysis of data based on their interviews or documents they had provided. To address the potential influence of these relationships, we discussed interpretations throughout the analytical process and continuously checked them against data from multiple participants, stakeholder groups, and documentary sources.

Focusing on stakeholders whose professional interests were affected is an important perspective to consider throughout the different phases of the development and implementation of complex interventions [29]. This approach, including the extensive collection of documents, also allowed for the inclusion of these perspectives without requiring these actors to participate directly in interviews.

The documentary material comprised a wide range of document types produced for different purposes and audiences and was analysed alongside interview data. Although this enabled a comprehensive exploration of power transfer during the implementation of self-admission, a more explicit examination of how document type and provenance shaped the representation of self-admission might have generated additional insights.

The documentary material and interviews reflected different time periods and stages of self-admission development and implementation. As the included models were introduced and evolved at different times, the material captured perspectives from multiple phases of implementation. Some findings may therefore be influenced by the specific implementation phase from which the data originated.

The included self-admission models differed in several aspects, including eligibility criteria, admission procedures, risk-management practices, and the degree of formal autonomy granted to patients. Although we considered these differences throughout the analysis, the study was not designed to compare specific models or evaluate the influence of individual model characteristics. Rather, we used the variation between self-admission models to broaden our understanding of power transfer across diverse self-admission contexts.

The study did not aim to examine how understandings of power transfer changed over time, nor to compare perspectives across stakeholder groups, countries, or self-admission models. Future studies could investigate whether differences in professional roles, organisational positions, national contexts, or model characteristics influence how power transfer, responsibility, and accountability are understood and enacted.

## 5. Conclusions

The implementation of psychiatric self-admission for individuals with severe mental illness illustrates how the structure of healthcare is challenged. Our findings raise important questions that healthcare providers may be occupied with, and which therefore need to be addressed, communicated, and taught about when implementing interventions defined by power transfer from healthcare providers to individuals with severe mental illness. Our findings illustrate the inherent and still existing inequalities and power relations embedded in psychiatry’s interaction with individuals with severe mental illness. Key aspects to consider for successful implementation and empowerment include the inherent power and accountability of the physician, views on what constitutes expert opinion, perspectives on safety and suicide risk and understanding formal and informal power relations. Empirical take-home messages for healthcare providers to consider before implementation in order to create meaningful empowerment include that patient safety is not only about preventing harm but about knowing when and how to safely place trust in shared responsibility. Taking back control, although it may be perceived to increase safety, may undermine both trust and autonomy. Formal power transfer may support change but is no guarantee for empowerment, as informal or invisible power structures may persist through implementation.

## Figures and Tables

**Table 1:** Overview of studied models of self-admission and examples of elements of formal power transfer.

Country	Model	Target Group	Contract	Length of Stay	Minimum Interval Between Self-Admissions *	Self-Administration of Medication	Risk Assessment During Admission
Denmark	Capital Region	Severe mental illness with a need for inpatient care	Individual	Up to 5 days	Flexible	Yes	Yes
Denmark	Central Region (Horsens)	Severe mental illness with a need for inpatient care	Individual	Individual	No	No	Yes
Norway	Western Norway Regional Health Authority (Jaeren)	Schizophrenia spectrum disorders with or without substance use disorder	Standardised	Up to 5 days	No	No	Yes
Norway	South-Eastern Norway Regional Health Authority (Follo)	Well-known patients with a recent history of inpatient care	Individual	Up to 5 days	14 days	No	Yes
Norway	Central Norway Regional Health Authority (Nidaros)	Schizophrenia spectrum disorders and bipolar disorders with extensive use of psychiatric services	Standardised	Up to 5 days	14 days	No	Yes
Sweden	Region Skåne	Individuals who self-harm and/or are at risk of suicide	Individual	Up to 4 days	No	Yes	No
Sweden	Region Stockholm	Severe mental illness with a need for inpatient care	Individual	Up to 4 days with possibility of individual adjustments	No	No	Yes

* The minimum mandatory time between self-admissions.

**Table 2:** Participants (n = 36).

Category		n
Country	Denmark	8
	Norway	7
	Sweden	21
Profession	Physician	12
	Nurse	13
	Psychologist	3
	Nurses’ aide	4
	Others	4
Role *	Regional Director of Psychiatry	2
	Head of Department	4
	Unit manager	4
	Chief Physician	3
	Project Manager	6
	Clinical Healthcare Staff	11
	Researcher	15

* Participants may hold multiple roles.

## Data Availability

Interviews and documents supporting findings of this study are not publicly available due to privacy restrictions.

## Supplementary Materials

The following supporting information can be downloaded at: https://www.mdpi.com/article/10.3390/healthcare14182909/s1, File S1: SRQR Checklist; File S2: Interview Guide; and File S3: Overview of Collected Documents. Please include this description in the Supplementary Files section.

## Abbreviations

The following abbreviations are used in this manuscript:

MRC	Medical Research Council
SRQR	Standards for Reporting Qualitative Research
REK	Norwegian Research Ethics Committees
EPM	Swedish Ethical Review Board
