# Supplementary material for: Stakeholder Perspectives on Power Transfer During Implementation of Psychiatric Self-Admission in Scandinavia: A Qualitative Study
Source: Healthcare (Basel). 2026 Sep 8;14(18):2909. doi: 10.3390/healthcare14182909 (PMC13606193; doi:10.3390/healthcare14182909)
Supplement: Supplementary file 1 [file healthcare-14-02909-s001.zip › Supplementary file S2.pdf]

Semi-structured interview guide

Exact questions depended on interaction during interviews. Estimated time: 45-90 minutes.

Example of questions relating to views, experiences, and approaches to intervention design and adaptation, including factors influencing how interventions were developed, adapted, and maintained.

What was your role in the development or adaptation? Who else was involved and what was their role? How were stakeholders involved? How were patients involved?

Can you describe how it was initiated to develop or adapt the model? When did this happen? How did you do it? Who were involved?

What are your experiences regarding how decisions on content and structure were taken? In what order? Who was involved and how? (Regarding for example inclusion/exclusion criteria, patient profile, clinical assessment, structure of admission, responsibilities and prerequisites, written basis)

Can you give some examples of details which were discussed, and thoughts or question marks or concerns/hopes related to those? What do you remember as most significant?

Were there any significant events that influenced the decisions in the development or adaptation process?

How well did the model agree with already existing interventions and values in healthcare?

Where was the model first used or tested? Was it evaluated? How? Was it appropriate?

Did it spread to other hospitals/regions/areas? How/why? Who decided?

Was it abolished anywhere? How/why? Who decided?

Has the model changed over time, if so how and why? Who has been involved in the process?

In hindsight: is there anything you think should have been done differently regarding the development, use or evaluation of the model? If so, what, and why? What were the main learnings?

Is there anything else you would like to add?

Do you have suggestions of any other person/s you think we should contact about this study?

Do you have any documentation you are willing to share with us regarding the model we have discussed? (reports, decisions, descriptions, contracts etc)
