# Supplementary material for: Stakeholder Perspectives on Power Transfer During Implementation of Psychiatric Self-Admission in Scandinavia: A Qualitative Study
Source: Healthcare (Basel). 2026 Sep 8;14(18):2909. doi: 10.3390/healthcare14182909 (PMC13606193; doi:10.3390/healthcare14182909)
Supplement: Supplementary file 1 [file healthcare-14-02909-s001.zip › Supplementary file S3.pdf]

Number	Country	Year	Documentation Type
1	Sweden	2015	Grant application
2	Sweden	2015	Training material
3	Sweden	2015	Contract template
4	Sweden	2014	Schedule
5	Sweden	2014	Educational material
6	Sweden	2012	Programme for scientific conference
7	Sweden	2017	Evaluation report
8	Sweden	2017	Evaluation report
9	Sweden	2014	Administrative decision
10	Sweden	2016	Status report
11	Denmark	2013	News article
12	Denmark	2016	Report
13	Denmark	2013	News article
14	Denmark	2013	News article
15	Denmark	2023	Administrative decision
16	Denmark	2024	Overview of self-admission models
17	Denmark	2013	News article
18	Sweden	2015	Grant application
19	Sweden	2019	Contract template
20	Sweden	2017	Contract template
21	Sweden	2023	Presentation
22	Sweden	2020	Email correspondence
23	Sweden	2020	Email correspondence
24	Sweden	2019	Email correspondence
25	Sweden	2018	Email correspondence
26	Sweden	2020	Email correspondence
27	Sweden	2022	Email correspondence
28	Sweden	2019	Email correspondence
29	Sweden	2022	Contract template
30	Denmark	2024	Contract template
31	Sweden	2024	Clinical routine
32	Sweden	2024	Referral form
33	Sweden	2024	Information material
34	Sweden	2024	Contract template
35	Norway	2024	Overview of self-admission models
36	Denmark	2017	PhD thesis
37	Denmark	2016	Evaluation report
38	Sweden	2020	Decision on clinical routine
39	Sweden	2021	Decision on clinical routine
40	Sweden	2020	Decision on clinical routine
41	Sweden	2020	Clinical routine
42	Sweden	2019	Clinical routine
43	Sweden	2019	Risk assessment
44	Sweden	2020	Risk assessment

45	Sweden	2020	Clinical routine
46	Sweden	2021	Clinical routine
47	Sweden	2016	Clinical routine
48	Sweden	2015	Report
49	Sweden	2016	Presentation
50	Norway	2020	Research article
51	Norway	2021	Report
52	Norway	2021	Research article
53	Denmark	2017	Information material
54	Denmark	2025	Overview of self-admission models
55	Denmark	2022	Information material
56	Denmark	2024	Guideline
57	Denmark	2022	Contract template
58	Denmark	2022	Contract template
59	Denmark	2022	Contract template
60	Norway	2025	Contract template
61	Norway	2025	Clinical routine
62	Norway	2011	Supporting documentation
63	Norway	2012	Supporting documentation
64	Norway	2008	Research article
65	Norway	2011	Presentation
66	Norway	2011	Conference programme
67	Norway	2024	Contract template
68	Norway	2016	Supporting documentation
69	Norway	2014	Policy document
70	Norway	2024	Clinical routine
71	Sweden	2025	Clinical routine
72	Sweden	2018	Supporting documentation
73	Sweden	2025	Clinical routine
74	Sweden	2019	Email correspondence
75	Sweden	2020	Email correspondence
76	Sweden	2017	Meeting notes
77	Sweden	2019	Meeting notes
78	Sweden	2017	Meeting notes
79	Sweden	2018	Meeting notes
80	Sweden	2018	Meeting notes
81	Sweden	2015	Clinical routine
82	Sweden	2016	Meeting notes
83	Sweden	2016	Meeting notes
84	Sweden	2022	Clinical routine
85	Sweden	2025	Report
86	Sweden	2024	Report
87	Sweden	2022	Report
88	Sweden	2015	Project plan
89	Denmark	2016	Survey response

90	Denmark	2024	Supporting documentation
91	Denmark	2018	Policy document
92	Denmark	2012	Policy document
93	Denmark	2021	Email correspondence
94	Denmark	2021	Supporting documentation
95	Denmark	2021	Supporting documentation
96	Norway	2019	Policy document
97	Norway	2021	Supporting documentation
98	Norway	2021	Official correspondence
99	Norway	2021	Supporting documentation
100	Norway	2021	Overview of rsearch material
101	Norway	2021	Email correspondence
102	Sweden	2022	Official directive
103	Sweden	2022	Report
104	Sweden	2021	Project plan
105	Sweden	2021	Meeting notes
106	Sweden	2021	Meeting notes
107	Sweden	2021	Meeting notes
108	Sweden	2021	Research article
109	Sweden	2021	Research article
110	Sweden	2021	Research article
111	Sweden	2021	Research article
112	Sweden	2021	Research article
113	Sweden	2021	Research article
114	Sweden	2021	Research article
115	Sweden	2021	Research article
116	Sweden	2021	Survey response
117	Sweden	2021	Research material overview
118	Sweden	2021	Research material overview
119	Sweden	2021	Evaluation report
120	Sweden	2021	Evaluation report
121	Sweden	2021	Research material overview
122	Sweden	2021	Evaluation report
123	Sweden	2021	Email correspondence
124	Sweden	2021	Email correspondence
125	Sweden	2021	Email correspondence
126	Sweden	2021	Email correspondence
127	Sweden	2021	Email correspondence
128	Sweden	2021	Email correspondence
129	Sweden	2021	Email correspondence
130	Sweden	2021	Email correspondence
131	Sweden	2021	Email correspondence
132	Sweden	2021	Email correspondence
133	Sweden	2021	Email correspondence
134	Sweden	2021	Research material overview

135	Sweden	2021	Evaluation report
136	Sweden	2021	Supporting documentation
137	Sweden	2021	Policy document
138	Sweden	2021	Email correspondence
139	Sweden	2021	Supporting documentation
140	Sweden	2021	Email correspondence
141	Sweden	2021	Guideline
142	Sweden	2021	Guideline
143	Sweden	2021	Official correspondence
144	Sweden	2021	Supporting documentation
145	Sweden	2021	Supporting documentation
146	Sweden	2021	Supporting documentation
147	Sweden	2021	Meeting notes
148	Sweden	2021	Legal document
149	Sweden	2021	Email correspondence
150	Sweden	2021	Legal document
151	Sweden	2021	Incident report
152	Sweden	2021	Incident report
153	Sweden	2021	Incident report
154	Sweden	2021	Incident report
155	Sweden	2021	Incident report
156	Sweden	2021	Incident summary
157	Sweden	2021	Legal Document
158	Sweden	2021	Official note
159	Sweden	2021	Supporting documentation
160	Sweden	2021	Supporting documentation
161	Sweden	2021	Supporting documentation
162	Sweden	2021	Supporting documentation
163	Sweden	2021	Interview questions
164	Sweden	2021	Survey response
165	Sweden	2021	Survey response
166	Sweden	2021	Survey response
167	Sweden	2021	Survey response
168	Sweden	2021	Survey response
169	Sweden	2021	Meetind agenda
170	Sweden	2021	Email correspondence
171	Sweden	2021	Report
172	Sweden	2021	Presentation
173	Sweden	2021	Meeting notes
174	Sweden	2021	Supporting documentation
175	Sweden	2021	Interview questions
176	Sweden	2021	Interview questions
177	Sweden	2021	Interview invitation
178	Sweden	2021	Interview invitation
179	Sweden	2021	Contact list

180	Sweden	2021	Survey response
181	Sweden	2021	Survey response
182	Sweden	2021	Survey response
183	Sweden	2021	Survey response
184	Sweden	2021	Survey response
185	Sweden	2021	Survey response
186	Sweden	2021	Survey response
187	Sweden	2021	Survey response
188	Sweden	2021	Survey response
189	Sweden	2021	Survey response
190	Sweden	2021	Survey response
191	Sweden	2021	Survey response
192	Sweden	2021	Survey response
193	Sweden	2021	Survey response
194	Sweden	2021	Survey response
195	Sweden	2021	Survey response
196	Sweden	2021	Survey response
197	Sweden	2021	Survey response
198	Sweden	2021	Survey response
199	Sweden	2021	Survey response
200	Sweden	2021	Survey response
201	Sweden	2021	Survey response
202	Sweden	2021	Survey response
203	Sweden	2021	Survey response
204	Sweden	2021	Survey response
205	Sweden	2021	Survey response
206	Sweden	2021	Survey response
207	Sweden	2021	Survey response
208	Sweden	2021	Survey response
209	Sweden	2021	Survey response
210	Sweden	2021	Survey response
211	Sweden	2021	Survey response
212	Sweden	2021	Data summary
213	Sweden	2021	Survey response
214	Sweden	2021	Data summary
215	Sweden	2021	Data summary
216	Sweden	2021	Contact list
217	Sweden	2021	Supporting documentation
218	Sweden	2021	Official correspondence
219	Sweden	2021	Official correspondence
220	Sweden	2021	Survey response
221	Sweden	2021	Survey response
222	Sweden	2021	Survey response
223	Sweden	2021	Survey response
224	Sweden	2021	Survey response

225	Sweden	2021	Survey response
226	Sweden	2021	Evaluation checklist
227	Sweden	2018	Service agreement
228	Sweden	2018	Service agreement
229	Sweden	2018	Service agreement
230	Sweden	2018	Service agreement
231	Sweden	2018	Service agreement
232	Sweden	2018	Service agreement
233	Sweden	2018	Service agreement
234	Sweden	2019	Service agreement
235	Sweden	2019	Service agreement
236	Sweden	2020	Service agreement
237	Sweden	2020	Service agreement
238	Sweden	2021	Service agreement
239	Sweden	2021	Service agreement
240	Sweden	2021	Service agreement
241	Sweden	2021	Service agreement
242	Sweden	2021	Service agreement
243	Sweden	2024	Service agreement
244	Sweden	2024	Service agreement
245	Sweden	2018	Service agreement
246	Norway	2026	Email correspondence
